# Clot Out, Flow Back: Large-Bore Aspiration Thrombectomy for Symptomatic Early Transjugular Intrahepatic Portosystemic Shunt (TIPS) Occlusion

**DOI:** 10.7759/cureus.116837

**Published:** 2026-09-24

**Authors:** Renato Abu Hana, Ryosuke Miyauchi, Yusuf Alzoubi, Grit A Adler, Vinicius Adami Vayego Fornazari

**Affiliations:** 1 Radiology, University of Florida College of Medicine – Jacksonville, Jacksonville, USA; 2 Internal Medicine, HCA Florida Blake Hospital, Bradenton, USA

**Keywords:** aspiration thrombectomy, balloon angioplasty, cirrhosis, interventional radiology, portal hypertension, shunt thrombosis, tips occlusion, transjugular intrahepatic portosystemic shunt

## Abstract

Thrombotic occlusion of a transjugular intrahepatic portosystemic shunt (TIPS) returns the patient to the portal-hypertensive state prior to shunt creation and requires early recognition and intervention. We describe a 45-year-old woman with metabolic dysfunction-associated steatohepatitis (MASH) cirrhosis who underwent TIPS placement for refractory ascites with subsequent clinical improvement. Approximately two months later, she presented with progressive anasarca, bilateral lower-extremity edema, abdominal distension, and diffuse abdominal pain. Computed tomography (CT) and Doppler ultrasound demonstrated complete in-stent shunt thrombosis, cirrhotic morphology, and moderate ascites. She was started on therapeutic heparin and underwent transjugular TIPS recanalization with large-bore mechanical aspiration thrombectomy followed by balloon angioplasty directed at the hepatic venous outflow segment. Completion venography demonstrated restored flow through the shunt without residual stenosis or obstruction; the portosystemic pressure gradient decreased from 21 to 13 mmHg. Her abdominal pain and manifestations of volume overload improved, and she was discharged on apixaban. This case demonstrates that single-session large-bore aspiration thrombectomy combined with balloon angioplasty can achieve technical, hemodynamic, and early clinical success in symptomatic early TIPS occlusion. Further studies are needed to determine its long-term efficacy and optimal role in TIPS revision.

## Introduction

Transjugular intrahepatic portosystemic shunt (TIPS) reduces portal pressure by creating a low-resistance channel between the portal and hepatic veins, decompressing the portal venous system. Portal hypertension drives life-threatening complications including ascites, variceal hemorrhage, hepatic hydrothorax, and hepatic encephalopathy [1]. TIPS is an established therapy when first-line medical treatment fails, and randomized trials support two principal indications: variceal bleeding unresponsive to standard therapy and refractory or recurrent ascites [1,2].

Despite continued refinement of TIPS technique and stent design, shunt dysfunction remains a persistent clinical challenge, most often resulting from thrombotic occlusion or progressive stenosis within the shunt [3]. Occlusion produces rebound portal hypertension, and patients redevelop ascites, abdominal distension, and other manifestations of the original indication. Endovascular revision of an occluded TIPS may involve mechanical thrombectomy or catheter-directed thrombolysis to restore patency, followed by balloon angioplasty and stent revision when an underlying stenosis or structural abnormality is identified [4].

Early TIPS dysfunction has no standardized temporal definition. We use the term to denote occlusion within the first three months of shunt creation, a window substantially earlier than the reported median time to first shunt dysfunction of approximately seven months [5]. Occlusion in this period is uncommon with the expanded polytetrafluoroethylene-covered stent grafts now standard for TIPS creation, and when it occurs it should prompt a search for a mechanical or technical explanation rather than attribution to pseudointimal hyperplasia alone, which characteristically develops later. This distinction derives from descriptive series rather than comparative data and is best regarded as a working framework [3,4].

This report describes successful endovascular recanalization of an occluded TIPS using a large-bore aspiration thrombectomy system combined with adjunctive balloon angioplasty. Mechanical thrombectomy has demonstrated high technical success in portal venous recanalization; in a series of 32 patients undergoing TIPS creation with thrombectomy for symptomatic acute noncirrhotic portal vein thrombosis, technical success was achieved in every patient [6]. This case illustrates a contemporary endovascular approach to symptomatic TIPS occlusion and contributes to the limited literature on large-bore aspiration thrombectomy for this indication.

## Case presentation

A 45-year-old woman with metabolic dysfunction-associated steatohepatitis (MASH) cirrhosis presented to the emergency department with three days of progressive anasarca, worsening bilateral lower-extremity edema, abdominal distension, and diffuse abdominal pain. She had undergone a TIPS procedure at an outside institution approximately two months earlier for refractory ascites, with subsequent clinical improvement and resolution of her diuretic and paracentesis requirement. Her liver-related history was notable for hepatic encephalopathy managed with lactulose and rifaximin. She had no prior history of portal or systemic venous thrombosis, no known thrombophilia, no active malignancy, and no recent surgery, hospitalization, or infection preceded the occlusion. A formal thrombophilia evaluation was not performed. She was receiving no anticoagulation and no prescription medications other than lactulose and rifaximin.

On the night before presentation, she developed worsening diffuse abdominal pain, rated 7/10 in severity, accompanied by persistent abdominal fullness and progressive lower-extremity edema, prompting emergency evaluation. She was afebrile and hemodynamically stable. Laboratory values at presentation and after intervention are summarized in Table 1. At presentation, before anticoagulation was initiated, the model for end-stage liver disease-sodium (MELD-Na) score was 14 [7], with an albumin of 2.6 g/dL, an international normalized ratio (INR) of 1.7, creatinine of 0.6 mg/dL, and total bilirubin of 0.5 mg/dL.

**Table 1:** Laboratory values at presentation and after transjugular intrahepatic portosystemic shunt (TIPS) recanalization. MELD-Na, model for end-stage liver disease-sodium

Laboratory parameter	At presentation	After TIPS recanalization	Reference range
Hemoglobin	8.9	9.6	12-16 g/dL
Platelet count	353	418	140-440 ×10³/µL
White blood cell count	6.27	6.24	4.5-11 ×10³/µL
Sodium	135	136	135-145 mmol/L
Potassium	3.9	4.5	3.4-4.5 mmol/L
Creatinine	0.6	0.7	0.51-0.95 mg/dL
Total bilirubin	0.5	0.4	0.2-1.0 mg/dL
Aspartate aminotransferase	69	50	14-33 U/L
Alanine aminotransferase	47	36	10-42 U/L
Alkaline phosphatase	170	152	35-104 U/L
Albumin	2.6	2.2	3.8-4.9 g/dL
International normalized ratio	1.7	2.4	0.9-1.1
MELD-Na score	14	17	-

Chest radiography showed no acute cardiopulmonary abnormality. Doppler ultrasonography of the liver with spectral and pulsed-wave analysis demonstrated occlusion of the mid and distal portions of the TIPS, with no detectable flow or spectral waveform, while the main portal vein remained patent. Subsequent computed tomography (CT) of the abdomen and pelvis demonstrated cirrhotic liver morphology and moderate ascites. The TIPS stent was well-positioned without kinking, though the shunt lumen showed no contrast opacification, a finding in keeping with thrombosis (Figure 1).

**Figure 1:**
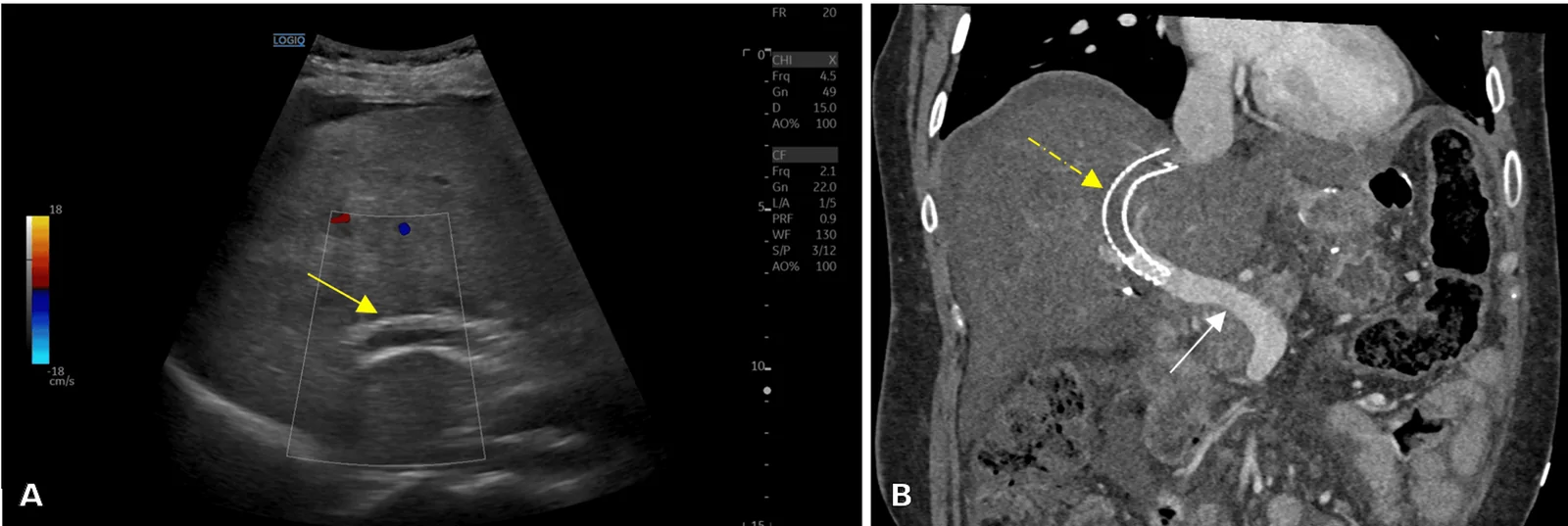
Doppler ultrasonography and contrast-enhanced CT demonstrating occlusion of the transjugular intrahepatic portosystemic shunt (TIPS). (A) Grayscale ultrasound with color Doppler interrogation of the TIPS (solid yellow arrow) showing no color flow signal within the shunt, in keeping with occlusion. (B) Coronal contrast-enhanced CT showing the TIPS stent, with cirrhotic hepatic morphology and moderate ascites. The dashed yellow arrow marks the non-opacified, thrombosed segment of the shunt lumen; the solid white arrow marks the adjacent, contrast-opacified, patent portal vein.

Interventional radiology was consulted for TIPS revision. The patient was started on a heparin infusion and underwent TIPS recanalization the following day after admission. Right internal jugular venous access was obtained under ultrasound guidance, and a 12 Fr GORE DrySeal Flex introducer sheath (W. L. Gore & Associates, Flagstaff, AZ) was placed. A 5 Fr Cobra catheter (Cook Medical, Bloomington, IN) was advanced into the inferior vena cava (IVC), where venography demonstrated no appreciable flow through the TIPS. The thrombosed segment was traversed with the catheter and a hydrophilic Glidewire (Terumo Corporation, Tokyo, Japan), allowing advancement of the catheter tip into the portal vein. Portal venography demonstrated complete occlusion of the shunt, with no flow from the portal venous system through the TIPS into the IVC (Figure 2).

**Figure 2:**
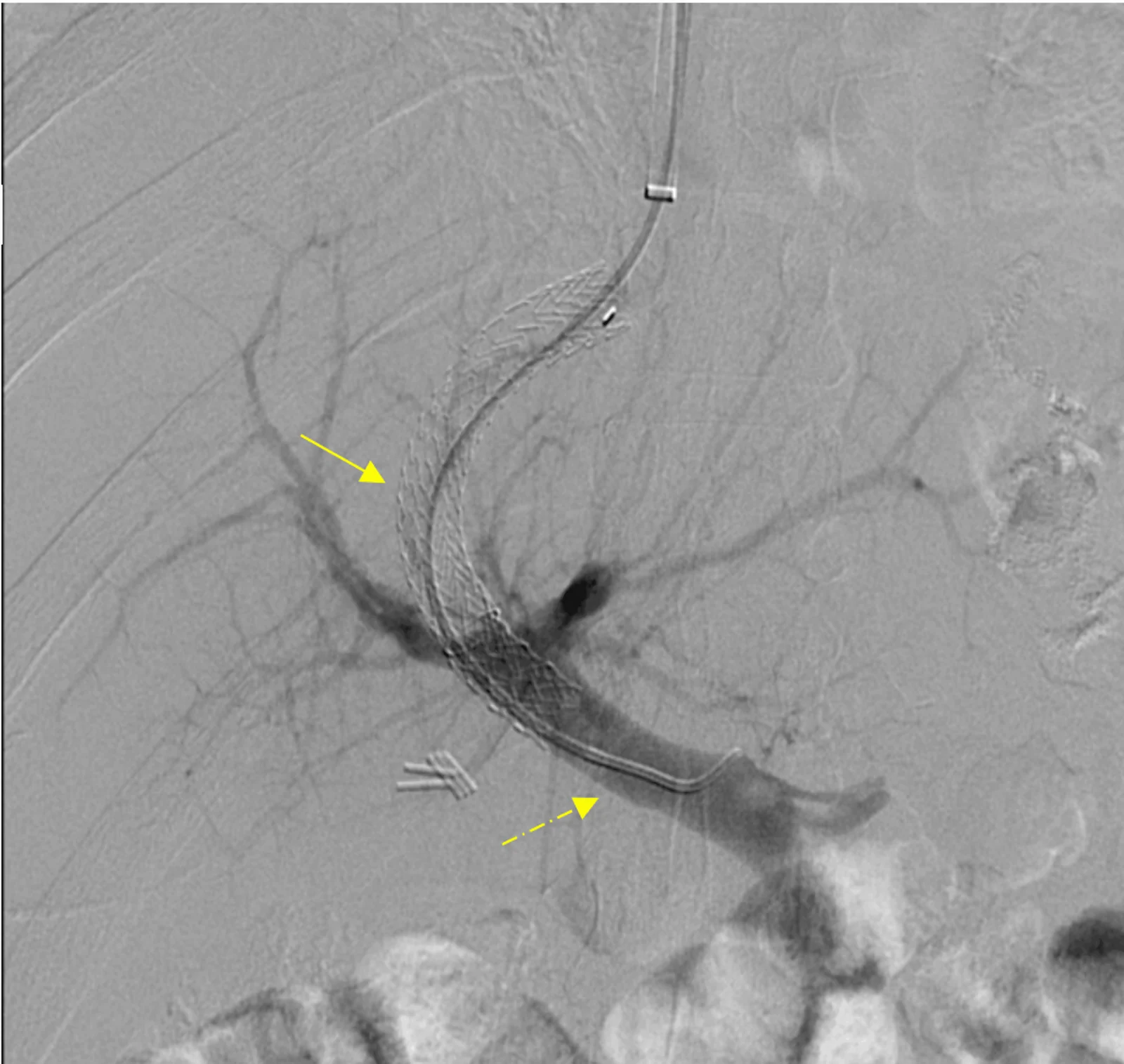
Procedural imaging during transjugular intrahepatic portosystemic shunt (TIPS) recanalization. Portal venography before thrombectomy showing complete occlusion of the TIPS with no flow into the inferior vena cava. The solid yellow arrow marks the site of occlusion within the TIPS stent; the dashed yellow arrow marks the patent portion of the portal vein filling with contrast.

The Glidewire was exchanged for an Amplatz Super Stiff guidewire (Boston Scientific Corporation, Marlborough, MA), and a 12 Fr Lightning Bolt 12 aspiration catheter (Penumbra, Inc., Alameda, CA) was advanced into the occluded shunt (Figure 3A). The baseline activated clotting time (ACT) was 220 seconds, reflecting the ongoing heparin infusion. After administration of 5,000 units of intravenous heparin, which raised the ACT to 265 seconds, mechanical aspiration thrombectomy was performed, removing thrombotic material. A further 2,000 units of heparin were administered empirically toward the end of the procedure, without a repeat ACT measurement, for a total intra-procedural dose of 7,000 units. (Figure 3B).

**Figure 3:**
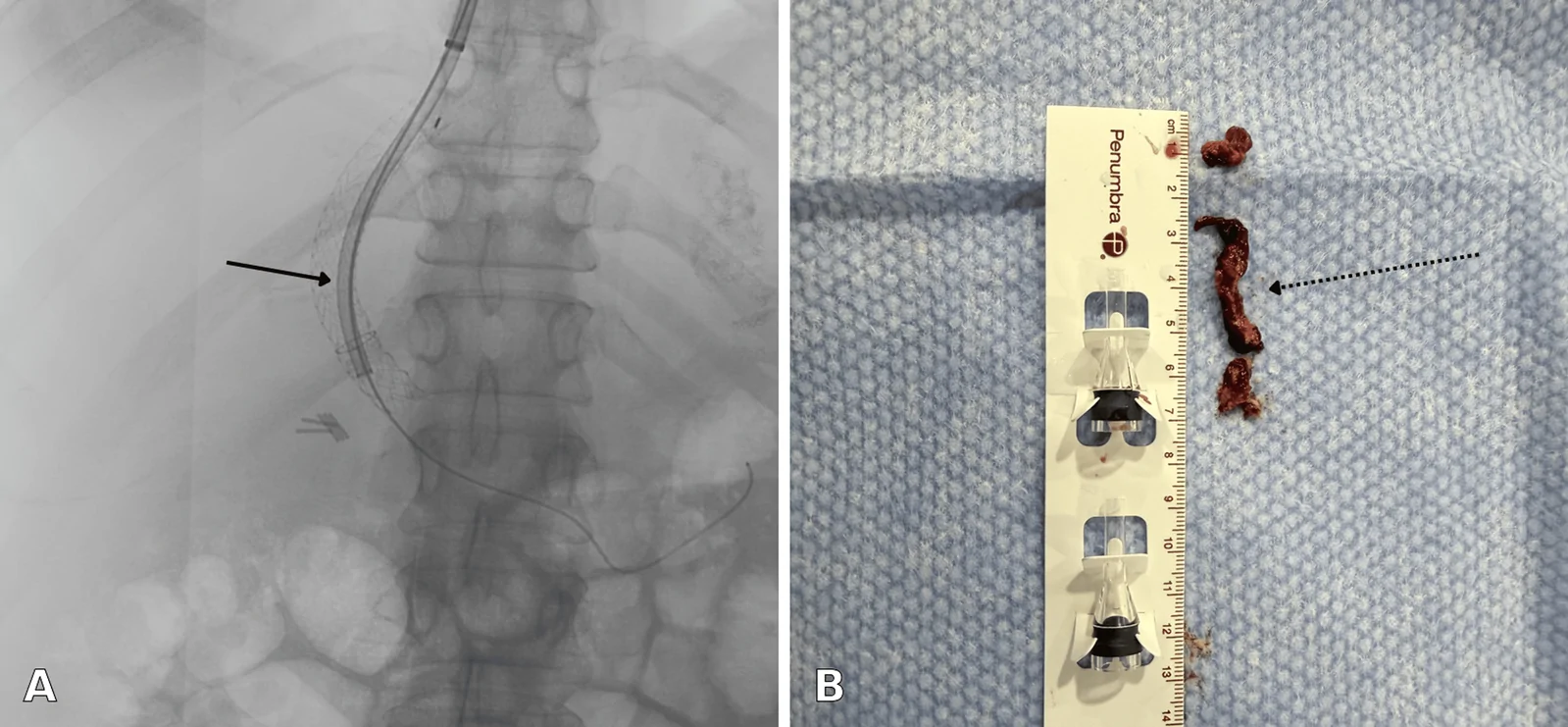
Aspiration thrombectomy and extracted thrombus. (A) Fluoroscopic image showing the Penumbra aspiration thrombectomy device positioned within the TIPS. The solid black arrow marks the device within the stent. (B) Gross photograph of the thrombotic material extracted from the occluded shunt, shown against a graduated ruler for scale. The dashed black arrow marks the extracted thrombus. The partial whitish discoloration of the specimen suggests an admixture of organized, chronic thrombus with more recently formed clot.

Balloon angioplasty was then performed across the TIPS with an 8 mm balloon followed by a 10 mm Mustang PTA balloon (Boston Scientific Corporation, Marlborough, MA), directed at the hepatic venous outflow segment (Figure 4A). Completion portal venography demonstrated restored flow through the TIPS without residual stenosis or obstruction (Figure 4B).

**Figure 4:**
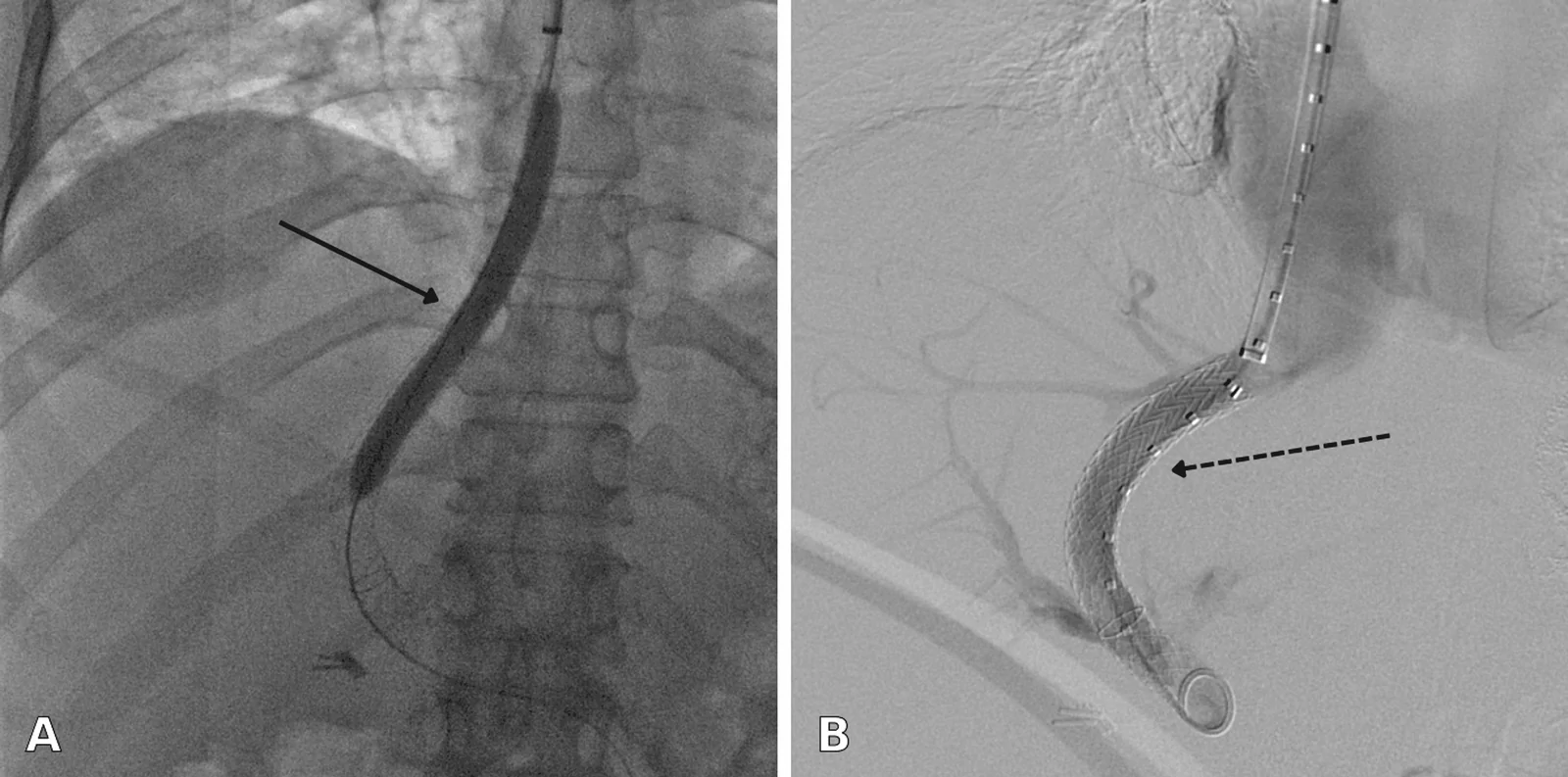
Balloon angioplasty and final portogram of the transjugular intrahepatic portosystemic shunt (TIPS). (A) Fluoroscopic image obtained during balloon angioplasty of the TIPS with an 8 mm balloon. The solid black arrow marks the balloon catheter positioned across the stent and hepatic venous outflow segment. (B) Final portal venogram demonstrating successful reconstitution of flow across the TIPS. The dashed black arrow marks the patent, well-apposed stent with restored antegrade flow.

Portal pressure was measured in the non-thrombosed segment of the main portal vein and systemic pressure in the IVC, using the same catheter and transducer system, with the transducer re-zeroed before each set of measurements. Both gradients were obtained at the same portal and caval sites during the same procedure, through the same transjugular access, under unchanged sedation and hemodynamic conditions. The portosystemic pressure gradient decreased from 21 mmHg before intervention to 13 mmHg after thrombectomy and angioplasty. All catheters and wires were removed, and no immediate procedural complications occurred.

The patient's abdominal pain and manifestations of volume overload improved over the following days, and postprocedural laboratory values are shown in Table 1. The heparin infusion was discontinued, and anticoagulation was transitioned to apixaban 5 mg twice daily, with discharge in stable condition. At one-month outpatient follow-up, she remained free of recurrent ascites, and surveillance Doppler ultrasonography demonstrated a patent shunt.

## Discussion

TIPS dysfunction restores the hemodynamic conditions the shunt was created to relieve, and the clinical presentation typically recapitulates the original indication. This patient had substantial improvement in refractory ascites after TIPS placement and no longer required diuretic therapy or large-volume paracentesis. Her return approximately two months later with anasarca, recurrent ascites, abdominal distension, and abdominal pain therefore raised immediate concern for shunt failure. Early TIPS dysfunction has no standardized temporal definition. We use the term to denote occlusion within the first three months of shunt creation. Occlusion in this period is uncommon with the expanded polytetrafluoroethylene-covered stent grafts now standard for TIPS creation, and when it occurs it should prompt a search for a mechanical or technical explanation rather than attribution to pseudointimal hyperplasia alone, which characteristically develops later; this distinction derives from descriptive series rather than comparative data and is best regarded as a working framework [3,4]. Proposed mechanisms of shunt failure include acute thrombus formation, pseudointimal hyperplasia, hepatic venous outflow stenosis, suboptimal stent position or length, stent migration, biliary-shunt communication, and prolapse of liver tissue into the shunt [8].

Doppler ultrasonography remains the first-line test for suspected TIPS dysfunction, particularly when the manifestations of portal hypertension recur. In this patient, CT first raised the possibility of thrombosis, and Doppler ultrasonography subsequently demonstrated absent flow and no detectable spectral waveform in the mid and distal shunt while the main portal vein remained patent, findings that establish occlusive rather than stenotic dysfunction. Doppler criteria are more reliable for the binary question of occlusion than for grading intermediate degrees of stenosis, and a normal-appearing study does not exclude a hemodynamically significant lesion. Venography with direct pressure measurement therefore remains necessary once intervention is contemplated, because it confirms both the anatomic and the hemodynamic significance of the lesion and determines the revision strategy [2].

Recanalization was performed transjugularly, exploiting the access used to create the shunt and preserving a direct working axis from the hepatic vein into the portal system. Large-bore aspiration thrombectomy was selected to remove obstructive thrombus rapidly, to re-establish a lumen within a single session, and to avoid a prolonged lytic infusion in a patient with cirrhosis, baseline coagulopathy, and portal hypertension, along with the associated monitoring burden. This rationale rests on the recognized bleeding risk of thrombolytic therapy in advanced liver disease [9] rather than on comparative data in TIPS occlusion, for which no studies directly comparing mechanical thrombectomy with catheter-directed thrombolysis are available. Mechanical aspiration also permits immediate interrogation of the underlying shunt once thrombus is cleared, so that a residual anatomic lesion can be identified and treated during the same procedure rather than after 12 to 24 hours of infusion.

Thrombus removal alone does not address the substrate that permitted thrombosis. Sequential 8 mm and 10 mm angioplasty was therefore performed across the shunt with attention to the hepatic venous outflow segment, a recognized site of TIPS dysfunction at which narrowing directly impedes outflow from the shunt and the segment most likely to have contributed to stagnation in this patient. Reported distributions of stenosis location after TIPS are heterogeneous and derive largely from the bare-metal stent era.

Completion venography demonstrated brisk transit of portal venous flow into the IVC without residual stenosis, and the portosystemic pressure gradient fell from 21 to 13 mmHg. The residual gradient warrants comment. Although a threshold below 12 mmHg is conventionally cited for control of ascites, that figure derives from hepatic venous pressure gradient measurements in untreated portal hypertension and translates imperfectly to intraprocedural portosystemic gradients, which are influenced by sedation, volume status, and the site of pressure sampling. The 8 mmHg reduction achieved here, together with restored shunt flow and prompt symptom resolution, was accepted as an adequate endpoint, and further shunt dilation was deliberately avoided given the competing risk of precipitating hepatic encephalopathy in a patient with an established history of it. Technical patency is an insufficient endpoint on its own and should be paired with an objective hemodynamic measurement and a documented clinical response [6,8].

Management should be individualized according to thrombus chronicity and extent, the presence of an underlying stenosis, access feasibility, and bleeding risk. Catheter-directed thrombolysis can be effective in acute thrombotic occlusion but requires prolonged infusion and carries added bleeding risk in advanced liver disease [10]. Balloon angioplasty alone is appropriate for focal stenosis but is insufficient when organized thrombus fills the shunt lumen. When thrombectomy unmasks persistent narrowing, particularly at the hepatic venous end, stent relining or extension is indicated. Recanalization occasionally requires alternative access, including transhepatic, transsplenic, transfemoral, or combined approaches, when the occluded shunt cannot be traversed from the transjugular route. Creation of a parallel TIPS is generally reserved for failed recanalization or an unsalvageable tract [8,9].

Antithrombotic management after TIPS revision is not standardized, and the evidence base must be distinguished from that for portal vein thrombosis in cirrhosis, where anticoagulation is supported by guideline recommendation [11]. No comparable evidence exists for thrombosis of the shunt itself: the available data on anticoagulation after TIPS are retrospective and heterogeneous [12], and address prophylaxis following shunt creation rather than secondary prevention after recanalization of an occluded shunt. This patient received therapeutic heparin from the time occlusion was suspected and was transitioned to apixaban at discharge, a strategy extrapolated from portal vein thrombosis practice rather than supported by shunt-specific data. Neither the agent, the duration, nor the incremental bleeding risk in patients with varices and thrombocytopenia has been defined for this indication.

The evidence supporting large-bore aspiration thrombectomy for occluded TIPS remains limited to case reports and small series, with substantial heterogeneity in device selection, access route, thrombus chronicity, adjunctive thrombolysis, angioplasty and stent relining, anticoagulation, and duration of follow-up. This report carries the limitations inherent to a single observation, including short follow-up and the absence of a comparator, and the underlying lesion that permitted thrombosis was inferred rather than directly demonstrated. It establishes the technical feasibility of single-session large-bore aspiration thrombectomy with adjunctive angioplasty for symptomatic early TIPS occlusion but cannot establish durability. A prospective study is needed to define patient selection, long-term shunt patency, and the optimal agent and duration of anticoagulation after TIPS revision.

## Conclusions

TIPS thrombosis can occur early, within a few months of an otherwise successful shunt placement. Recurrence of ascites, anasarca, or abdominal pain after initial improvement warrants prompt evaluation for shunt occlusion. In this patient, large-bore aspiration thrombectomy with targeted balloon angioplasty achieved complete single-session recanalization, with restored shunt flow and symptomatic improvement at one month; durability is a separate question, governed by the residual outflow substrate and post-revision anticoagulation. The patient was not receiving anticoagulation at the time of the thrombotic event, and while a single observation cannot establish that anticoagulation would have prevented occlusion, the case highlights the uncertainty surrounding prophylactic anticoagulation after TIPS rather than supporting its routine use. Prospective evaluation is needed to define whether, in whom, and for how long anticoagulation should be given after shunt creation or revision.
